# The Role of Pro-Inflammatory Cytokines in the Pathogenesis of Cardiovascular Disease

**DOI:** 10.3390/ijms25021082

**Published:** 2024-01-16

**Authors:** Hannah Zhang, Naranjan S. Dhalla

**Affiliations:** 1 Institute of Cardiovascular Sciences, St. Boniface Hospital Albrechtsen Research Centre, Winnipeg, MB R2H 2A6, Canada; 2 Department of Physiology and Pathophysiology, Rady Faculty of Health Sciences, College of Medicine, University of Manitoba, Winnipeg, MB R3E 0J9, Canada

**Keywords:** cardiovascular disease, inflammation, cytokines, cardiomyopathy, cardiac remodeling, ischemic heart disease, heart failure

## Abstract

With cardiovascular disease (CVD) being a primary source of global morbidity and mortality, it is crucial that we understand the molecular pathophysiological mechanisms at play. Recently, numerous pro-inflammatory cytokines have been linked to several different CVDs, which are now often considered an adversely pro-inflammatory state. These cytokines most notably include interleukin-6 (IL-6),tumor necrosis factor (TNF)α, and the interleukin-1 (IL-1) family, amongst others. Not only does inflammation have intricate and complex interactions with pathophysiological processes such as oxidative stress and calcium mishandling, but it also plays a role in the balance between tissue repair and destruction. In this regard, pre-clinical and clinical evidence has clearly demonstrated the involvement and dynamic nature of pro-inflammatory cytokines in many heart conditions; however, the clinical utility of the findings so far remains unclear. Whether these cytokines can serve as markers or risk predictors of disease states or act as potential therapeutic targets, further extensive research is needed to fully understand the complex network of interactions that these molecules encompass in the context of heart disease. This review will highlight the significant advances in our understanding of the contributions of pro-inflammatory cytokines in CVDs, including ischemic heart disease (atherosclerosis, thrombosis, acute myocardial infarction, and ischemia-reperfusion injury), cardiac remodeling (hypertension, cardiac hypertrophy, cardiac fibrosis, cardiac apoptosis, and heart failure), different cardiomyopathies as well as ventricular arrhythmias and atrial fibrillation. In addition, this article is focused on discussing the shortcomings in both pathological and therapeutic aspects of pro-inflammatory cytokines in CVD that still need to be addressed by future studies.

## 1. Introduction

Cardiovascular disease (CVD) is the most prominent cause of morbidity and mortality across the world. In fact, the World Health Organization (WHO) estimated nearly 18 million global deaths due to CVD in 2019 [1]. In the North American continent, CVD accounted for 36.4 million years of life lost and 4.5 million years lived with disability between 2000 and 2019 [2]. In the United States, CVD had an economic burden of over 32 billion dollars in 2016 [3]. In view of its high prevalence and cost, there is still an urgent need to discover the pathophysiology of CVD, and there is much to understand about its therapeutics and management. Although there is a diverse array of heart disease etiologies, the involvement of aberrant inflammatory processes seems to be a common link between different types of CVDs.

It is pointed out that inflammation is a key component of the immune system of the body, as it is involved in protection against infection and repair of tissue damage. While normally a tightly controlled response, inflammation can also cause severe disease and even mortality when it goes rogue. In fact, inflammation is a biphasic phenomenon that exerts cellular protection during early phases and induces cellular damage during late phases. Several studies over the past few decades have revealed that the immune system and inflammation play an important role in heart disease pathology. Many cell types are involved in regulating inflammation, including the key immune players of macrophages, neutrophils, and lymphocytes [4]. In order to achieve a coordinated response, small proteins called cytokines are secreted by various cell types in the form of signaling and communication molecules, but the cytokine network is very complex and has built-in redundancies that have been outlined by some investigators [5,6]. It is noteworthy that cytokines act in an autocrine, paracrine, or endocrine fashion, and the primary role of cytokines is to modulate the proliferation of immunoregulatory cells in a pro-inflammatory or anti-inflammatory manner and affect targeted cell migration via chemotaxis. Some investigators have postulated that the balance between the opposing actions of pro- and anti-inflammatory cytokines is what keeps tissues in a healthy and regulated state [7]. In 1996, Seta et al. [8] proposed “the cytokine hypothesis”, which suggested that the progression of heart failure is manifested due to the actions of excessive endogenous cytokines. Some of the known pro- and anti-inflammatory cytokines relevant to heart disease are shown in Table 1 [7,9,10,11,12,13,14,15,16,17,18,19,20,21,22,23,24,25,26,27,28,29,30]. Anti-inflammatory cytokines function primarily as regulators of pro-inflammatory activities to limit tissue self-damage, and these are considered responsive to the dynamic changes in inflammatory response. Such interactions are extremely complex, and it is only recently some efforts have begun to map the intricate cytokine network.

In view of the opposing effects of different cytokines in the pathogenesis as well as therapeutics of CVD, it is becoming evident that a balance of pro-inflammatory and anti-inflammatory cytokines is essential for maintaining cardiovascular health. Although the role of both types of cytokines in cardiac dysfunction has been extensively reviewed [14,15,17,21,25,30], the mechanisms of their exact involvement in the inflammatory processes in various CVDs are not fully understood. Since there is a great potential for developing an appropriate therapy for the treatment of heart disease by identifying some cytokines as a molecular target, this article was undertaken to briefly provide updated state-of-the-art information on the general characteristics of several pro-inflammatory cytokines. Furthermore, it is intended to discuss the role of different pro-inflammatory cytokines in the pathophysiology of several types of CVDs, including atherosclerosis, myocardial infarction, ischemia-reperfusion injury, hypertension, cardiac hypertrophy, heart failure, atrial fibrillation, and ventricular arrhythmias, as well as different types of cardiomyopathies. The participation of pro-inflammatory cytokines in the development of cardiac fibrosis and apoptosis, as well as their relationship with other pathogenic factors, such as oxidative stress and intracellular calcium overload, will be described.

## 2. General Characteristics of Pro-Inflammatory Cytokines

This review is focused on the role of pro-inflammatory cytokines in heart diseases, with a discussion on the findings and shortcomings of pharmacotherapies developed in this field. The heterogeneous nature of cytokines also makes them important both in systemic circulation and localized to the myocardial and vascular tissue. A brief overview of some prominent pro-inflammatory cytokines relevant to heart disease is presented below.

### 2.1. Interleukin-6 (IL-6)

IL-6 is an important cytokine implicated in many heart diseases and has a diverse array of functions. While classically considered a pro-inflammatory cytokine, IL-6 also has anti-inflammatory properties, making its mechanisms of action in disease pathophysiology very complex. In CVD, IL-6 is produced by macrophages, monocytes, endothelial cells, vascular smooth muscle cells, and fibroblasts [31,32]. Myeloid cells also produce IL-6, along with IL-1β and tumor necrosis factor (TNF)α, upon toll-like receptor (TLR) activation. This initiates a massive positive feedback loop and rapidly amplifies IL-6 production by six orders of magnitude [33]. IL-6 binds to the membrane-bound IL-6 receptor (IL-6R), which is present in several cells, including endothelial cells and leukocytes, to activate its classical signaling cascade. The IL-6/IL-6R complex associates with glycoprotein 130 kDa (gp130), dimerizes, and activates intracellular signaling pathways, including the Janus kinase/signal transducer and activator of transcription (Jak/Stat) pathway [31]. Soluble IL-6R is important in trans-signaling, as it allows IL-6 to activate cells that do not express IL-6R on their membranes [31]. Classical IL-6 signaling is associated with anti-inflammatory properties, while trans-activation is associated with pro-inflammatory qualities [13,33]. The pro-inflammatory actions of IL-6 include T-cell apoptosis inhibition, inflammatory cell recruitment, and inhibition of regulatory T-cell differentiation [34]. IL-6 is an important mediator for acute phase reactions, and its levels are correlated with C-reactive protein (CRP) levels [10]. As a result, both IL-6 and CRP are used clinically as biomarkers of inflammation [35].

### 2.2. Tumour Necrosis Factor-α (TNFα)

TNFα was one of the first pro-inflammatory cytokines linked to heart diseases. In fact, both cardiac myocytes and macrophages produce TNFα, which can work in an autocrine and paracrine manner to perpetuate inflammation [36]. Type 1 T-helper (Th1) cells also produce this cytokine. TNFα exists both in a transmembrane and a soluble form, which, like IL-6R, allows this cytokine to diversify its functions. The production of TNFα sets off a cascade of pro-inflammatory signaling, which has both protective and destructive effects on tissue. The vastly varied nature of TNFα signaling can, in part, be explained by the presence of TNFα receptors (TNFR) on almost all cell types. There are two main types of receptors for this cytokine, namely TNFR1 (p55) and TNFR2 (p75), which differ in their intracellular domains, thereby causing different signaling cascades [37]. Activation of TNFR1 is associated with deleterious effects, while TNFR2 stimulation is involved in protective mechanisms [37]. Interestingly, TNFRs can shed their extracellular domains under certain stimuli, including excess circulating TNFα, although it is unclear if this is an adaptive response to neutralize serum TNFα or works to enhance TNFα’s activity [38]. One of the primary roles of TNFα in the context of myocardial tissue is inducing cell apoptosis in both myocytes and endothelial cells [36]. It also contributes to oxidative stress by upregulating nitric oxide production, plays a role in calcium dysregulation, and recruits neutrophils and macrophages [22,38,39]. An important endogenous modulator of TNFα is its shedded soluble receptor sTNF-RII [40], and TNFα’s secretion is inhibited by the anti-inflammatory cytokine IL-10 [41]. Additionally, TNFα is implicated in angiogenesis and thrombogenesis, which are important in heart disease development [42].

### 2.3. Interleukin-1 (IL-1) Family

The IL-1 family consists of 11 ligands and 10 receptors spread over three subfamilies: IL-1 subfamily, IL-18 subfamily, and IL-36 subfamily [43]. The IL-1 family is important in the innate immune response and contributes to destructive inflammation. With so many ligands and receptors involved, this large group of cytokines contains primarily pro-inflammatory components but also has elements that antagonize and regulate inflammation [44]. IL-1α, IL-1β, and IL-18 are the primary pro-inflammatory cytokines implicated in heart disease pathophysiology. IL-1α is constitutively expressed in epithelial and mesenchymal cells (including the myocardium) in healthy conditions and is released when a cell is injured or dies, while IL-1β is upregulated in disease and is the main circulating type of IL-1 [43,45]. As a result, IL-1β has become one of the most widely studied cytokines to determine if it is a viable therapeutic target for a variety of heart diseases. Like the previous cytokines discussed, both IL-1β and IL-18 are produced primarily by monocytes and macrophages but also by neutrophils and accumulate inside the cytoplasm until activation by caspase-1 and nucleotide-binding domain and leucine-rich repeat pyrin containing protein-3 (NLRP3) [43,46,47]. IL-1β mainly induces inflammation through IL-6 signaling and acute phase proteins such as CRP [47]. In contrast, while also part of the IL-1 family, IL-37 and IL-38 reduce the levels of these proinflammatory cytokines [46]. Additionally, the endogenously produced IL-1 receptor antagonist (IL-1Ra), commercially known as anakinra, blocks IL-1α and IL-1β’s biological activity by binding to their receptor to prevent their association [19].

### 2.4. Interferon-γ (IFN-γ)

IFN-γ, the only member of the type II IFN family, has been implicated in many heart diseases. This cytokine is produced by macrophages, CD4+ and CD8+ T-lymphocytes, and natural killer (NK) cells and is therefore involved in both innate and adaptive immune responses [48]. IFN-γ production can be stimulated by IL-18 and IL-2 [49,50]. When cleaved to its active form, IFN-γ induces signaling via the Jak/Stat pathway and stimulates low-density lipoprotein (LDL) accumulation in macrophages [48]. IFN-γ also stimulates scavenger receptor expression on smooth muscle cells and promotes their migration into the arterial intima, and increases the expression of adhesion molecules on activated endothelial cells. IFN-γ promotes macrophage polarization towards the pro-inflammatory M1 phenotype, which becomes important in many heart diseases.

### 2.5. Transforming Growth Factor-β (TGF-β)

Although widely known for its anti-inflammatory properties, TGF-β is a pleiotropic cytokine that plays an important role in inflammation and cellular damage in heart disease. TGF-β has three isoforms, but TGF-β1 is by far the most widely studied in human physiology. While most cells in the heart can produce TGF-β, cardiomyocytes and infiltrating macrophages appear to be a major source in heart disease; however, activated fibroblasts, vascular endothelial cells, mast cells, and lymphocytes also produce TGF-β in certain situations [20]. TGF-β’s activities are closely linked to T-cell differentiation, homeostasis, and tolerance. Under IL-6 stimulation, TGF-β promotes T helper 17 (Th17) cell, NK cell, and T regulatory (Treg) cell differentiation [21,51]. TGF-β is also a chemo-attractant for neutrophils, monocytes, and leukocytes and plays a role in macrophage polarization towards an M2 phenotype [20]. It is thus critical in the transition between tissue inflammation and repair.

### 2.6. Granulocyte Colony-Stimulating Factor (G-CSF)

In humans, G-CSF is primarily made in cardiomyocytes, monocytes, fibroblasts, and endothelial cells [22]. Its most notable role in cardiac pathophysiology involves the stimulation of neutrophil proliferation and differentiation from monocytes [52]. Additionally, G-CSF appears to protect vascular endothelial cells and cardiomyocytes from apoptosis [22].

### 2.7. Granulocyte-Macrophage Colony-Stimulating Factor (GM-CSF)

GM-CSF is produced mainly by T-cells but can also be secreted by epithelial and endothelial cells, as well as fibroblasts [22,53]. This cytokine has a diverse range of roles in inducing inflammation, including stimulating the survival, differentiation, and proliferation of neutrophils, eosinophils, macrophages, dendritic cells, and mast cells [22].

### 2.8. Interleukin-2 (IL-2)

While not as well-studied as some other cytokines, IL-2 has been experimentally implicated in heart disease. IL-2 is known for its role in Treg cell development and survival, which is important in tolerance and muting the immune response [24,54]. However, IL-2 has dual roles in inflammation as it is important in promoting the proliferation and differentiation of effector T-cells when secreted by a naïve T-cell activated by an antigen-presenting cell [24]. Clinically, it has primarily been used in the field of oncology, but it has known cardiotoxic effects, particularly at high doses [55,56].

### 2.9. Interleukin-17 (IL-17)

Only recently have researchers begun understanding the role that IL-17 plays in human disease pathophysiology. The primary source is a group of CD4+ T helper cells characterized by their expression of IL-17, termed Th17 cells, although other T lymphocytes and myeloid cells have also been shown to express this cytokine in lower amounts. IL-17 acts primarily through nuclear factor-κB (NF-κB) and mitogen-activated protein kinase (MAPK) pathways, resulting in upregulation of pro-inflammatory gene transcription and stabilization of target mRNA transcripts. Non-hematopoietic cells, such as fibroblasts, are the primary targets of IL-17 [57]. IL-18 can stimulate the release of IL-17 [58]. 

## 3. Inter-Relationships of Inflammation with Other Pathogenic Mechanisms

Part of the complexity of inflammation is its involvement in several other destructive signaling pathways. It is now widely recognized that in addition to inflammation, two major pathophysiologic mechanisms, oxidative stress and calcium (Ca^2+^) mishandling, are intimately involved in the development of cellular dysfunction [59,60,61]. Although the effectiveness and inter-relationships of these three mechanisms for inducing heart disease are not fully understood, these processes have been shown to modify cardiac gene expression, myocardial metabolism, and the activities of different proteases [62,63,64,65,66,67,68,69,70,71]. A broad summary of the links between oxidative stress and calcium mishandling to inflammation is as follows.

### 3.1. Oxidative Stress

Oxidative stress and inflammation are often considered linked processes, but the classic chicken-and-egg question remains: which comes first? Oxidative stress involves the excessive accumulation of reactive oxygen species (ROS) as a result of an imbalance between ROS production and elimination. Normally, ROS are mainly produced by the mitochondria in cellular respiration by enzymes such as NADPH oxidase and are eliminated by antioxidant mechanisms, including superoxide dismutases and peroxidases [72,73,74]. With chronic overproduction of ROS, cellular damage and mutations can occur, leading to cellular damage and disease [74]. In relation to inflammation, when immune cells are recruited to sites of damage, there is a “respiratory burst” in which there is an increase of uptake in oxygen, leading to ROS production and accumulation [74]. Conversely, ROS are also considered to be an inflammatory stimulus and can recruit immune cells and induce secretion of pro-inflammatory cytokines [73]. With each aggravating the other, oxidative stress and inflammation can create a vicious cycle of tissue destruction. Nevertheless, extensive work needs to be carried out to understand the nature of the interaction of different pro-inflammatory cytokines with oxidative stress at various signal transduction pathways.

### 3.2. Calcium Mishandling

Ca^2+^ homeostasis is integral to numerous important physiological and pathophysiological processes. In the heart it is involved in excitation-contraction coupling, as it plays a role both in the electrical pacemaker and physical pumping properties of the heart [75]. Ca^2+^ is also important in the relaxation of the myocardium following contraction, as excessive intracellular Ca^2+^ will lead to the inability of the heart to relax. It should be noted that hypocalcemia in circulation is related to different types of cardiomyopathies and heart failure, whereas hypercalcemia leads to the calcification of heart valves and vessels [76]. Under disease conditions associated with intracellular Ca^2+^ overload, cells can die and release different proteins, which may lead to an inflammatory response [77]. Conversely, pro-inflammatory cytokines also play a role in Ca^2+^ homeostasis via the regulation of ion channels, and thus, these molecules may remodel the electrical activity of the heart [77]. Although oxidative stress has been demonstrated to produce intracellular Ca^2+^ overload [60,64], it is not clear whether such an effect of inflammation is mediated through the participation of oxidative stress. In view of the profound actions of Ca^2+^ in the regulation of various processes such as gene expression, myocardial metabolism, cellular permeability, protease activation, and cellular death, it appears that Ca^2+^ handling abnormalities in the myocardium may play a critical role in the development of CVD as a consequence of oxidative stress and inflammation.

## 4. Inflammatory Cytokines in Heart Disease

### 4.1. Ischemic Heart Disease

Ischemic heart disease (IHD) comprises a spectrum of diseases that are primarily related to the coronary arteries. It is the most common cause of death globally and is associated with significant morbidity [78]. While traditionally considered synonymous with atherosclerotic disease, we are now beginning to understand the complexities in its pathophysiology beyond just chronic plaque buildup. It is pointed out that inflammation is one branch of the intricate pathway network that has started gathering traction among several investigators. 

#### 4.1.1. Atherosclerosis

Atherosclerosis involves the proliferation of smooth muscle cells, cholesterol deposition in the arterial walls, and infiltration of monocytes. These processes result in lesions that narrow blood vessels and restrict distal flow [79]. Being one of the most common diseases in the world, the link between atherosclerosis and pro-inflammatory cytokines has been quite well-characterized. Vascular smooth muscle cells are known to be a source of IL-6 [80], which has been shown to play an important role in atherosclerosis. In fact, IL-6 injection has been reported to cause significant increases in other pro-inflammatory cytokines, including IL-1β and TNFα, and early development of atherosclerotic lesions [81]. IL-6 mRNA transcripts and proteins were observed to be expressed in the atherosclerotic plaques and arterial walls in humans and rodents at higher rates than non-atherosclerotic artery tissue [79,82,83,84]. Apolipoprotein E (ApoE) and IL-6 double knockout mice were found to have significantly larger and more calcified arterial lesions and no difference in hypercholesterolemia compared to just ApoE knockout animals, and it was suggested that IL-6 expression is important in the formation of fibrous plaque of atheroma [85]. These findings were replicated by Schieffer et al. [86], who showed that IL-6/ApoE double knockout mice have significantly increased atherosclerotic lesions and proposed that IL-6 is important in vascular development, lipid homeostasis, and plaque inflammation. 

Another study showed that IL-6 expression in plaques increases with age and lesion intensity in the isolated aortic rings of ApoE knockout mice [83]. While initially seeming contradictory, these studies reinforce the concept that IL-6 plays dual roles, being anti-inflammatory in some circumstances and pro-inflammatory in others. Clinically, higher circulating levels of IL-6 and C-reactive proteins were associated with increased all-cause mortality [87,88], and 6-month mortality was reduced in patients with high IL-6 levels who were given dalteparin therapy [88]. Patients with type 2 diabetes (T2DM) and macrovascular atherosclerosis showed higher levels of circulating IL-6 compared to patients with atherosclerosis and no diabetes, and in combination with levels of TNFα, were better at predicting atherosclerosis development in T2DM patients [89]. Patients with atherosclerosis also had higher levels of IL-6 in the blood than patients without atherosclerosis [89,90], and patients with unstable angina showed higher levels of circulating IL-6 than patients with stable angina [91,92]. IL-6 levels were also higher in patients with ischemic heart failure compared to simple coronary artery disease [93]. Unstable coronary disease patients with complicated hospital courses had higher IL-6 levels than patients with uneventful courses [94]. Coronary artery disease patients who were given an aerobic exercise program in rehabilitation showed reduced circulating levels of pro-inflammatory cytokines, including IL-6 [95]. Overall, it appears that while the complete lifetime absence of IL-6 leads to vascular maladaptation and reduced atherosclerosis in animal models, increased circulating IL-6 levels in patients are associated with the development and severity of atherosclerotic disease.

While TNFα was found in both human and murine arterial walls with atherosclerotic and other lesions [42,90,96,97,98], its absence did not reduce lesion size in mice; however, the absence of lymphotoxin-α, a cytokine with homology to TNFα, reduced atherosclerosis [96]. In isolated rat hearts, ischemia alone was sufficient in increasing the TNFα levels in a time-dependent manner [99]. Human carotid atheromatous plaques were observed to have activated macrophages that produce TNFα and IL-1 [100]. Both platelet-activating factor and oxidized low-density lipoprotein (oxLDL), which are present in atherosclerotic plaques, promoted TNFα production in peripheral blood [101]. In cultured human endothelial and smooth muscle cells, TNFα induced the expression of macrophage colony-stimulating factor (M-CSF), which caused the proliferation and differentiation of monocytes and is important for macrophage survival and maturation [102]. Blood TNFα levels were higher in patients with atherosclerosis compared to those without [90] and higher in ischemic heart failure patients compared to coronary artery disease patients [93]. Unexpectedly, the absence of the TNFα p55 receptor in mice was found to be associated with increased atherosclerotic lesion development compared to wildtype mice, indicating the TNFα receptor is protective against atherosclerosis [103]. On the other hand, it is noteworthy that a clinical study involving healthy men showed that plasma TNFα levels are associated with early atherosclerotic lesion development and CVD risk factors, including blood pressure, dyslipidemia, and indices of insulin resistance [104]. 

Both IL-1α and β are secreted by different cell types in the vascular wall, including smooth muscle cells and endothelial cells. When cultured with monocyte-derived IL-1 or human recombinant IL-1, smooth muscle cells produced biologically active IL-1 [105], indicating the presence of this cytokine can stimulate its own expression. Endothelial cells were also shown to produce IL-1 when given inflammatory stimuli such as endotoxin and TNFα [106,107]. IL-1 was observed to be crucial in initiating atherosclerosis in ApoE-deficient mice, as the administration of IL-1Ra significantly reduced fatty streak formation on arterial walls [108,109]. IL-1Ra knockout mice fed an atherogenic diet also had a trend of increased foam cell adhesion area compared to their wildtype littermates, and overexpressing IL-1Ra in LDLR knockout mice was observed to decreased foam cell adhesion area [110]. Aortic sinus atherosclerotic lesions were significantly reduced in ApoE and IL-1β double knockout mice compared to ApoE deficiency alone [111]. IL-1β treatment potentiates vasospasm in pig arteries and caused coronary artery lesions [112]. Clinically, IL-1Ra can be measured as a proxy for IL-1β, and its levels are higher in patients with unstable coronary disease who have a complicated hospital course compared with an uneventful course [94]. Higher IL-1β levels are found in the pericardial fluid of patients with ischemic heart disease compared to valvular and congenital heart disease groups [113]. Clinical trials have been performed to try and target IL-1 to reduce atherosclerotic risk. A randomized control trial called CANTOS (Canakinumab Anti-inflammatory Thrombosis Outcome Study) with over 10 thousand patients with previous myocardial infarction received different doses of canakinumab, a monoclonal antibody for IL-1β, or placebo [114]. The highest dose of the drug administered every 3 months resulted in significantly fewer cardiovascular events than placebo, and this result was found to be independent of lipid levels [115]. Canakinumab also reduced the total amount of serious cardiovascular events long-term, with a median follow-up of ~3.7 years [116]. These results suggest the potential of anti-inflammatory therapy targeted at IL-1β for reducing cardiovascular risk.

Previous results on IFN-γ’s role in atherosclerosis have yielded inconclusive findings, as the cytokine appeared to have both pro- and anti-atherogenic properties. Specifically, IFN-γ promoted vascular cell adhesion molecule-1 (VCAM-1) expression on endothelial cells [117] and increased scavenger receptor production in smooth muscle cells in vitro [118], which are pro-atherogenic properties but suppressed the LDL receptor pathway in macrophages [119] and inhibited smooth muscle cell proliferation [120], which are anti-atherogenic features. However, more recent animal models and human studies have provided evidence of the pro-atherogenic nature of this cytokine. In the ApoE knockout mouse model, IFN-γ receptor knockout induced a large reduction in atherosclerotic lesion size and a reduction in lipid accumulation, suggesting IFN-γ promotes and modifies both atherosclerotic lesions and plasma lipoprotein profiles [121]. In LDLR and IFN-γ double deficient mice, atherosclerotic lesion size was also significantly reduced compared to LDLR knockout alone but was independent of lipoprotein profiles [122]. Locally activated T-cells were detected in human atherosclerotic plaques, with IFN-γ being detected around the lymphocytes, indicating paracrine secretion in the lesion [123]. This pro-inflammatory cytokine was also detected by immunohistochemistry in human plaques [124]. IFN-γ inducible factors are found to be differentially expressed by atheroma-associated cell types, including ECs, SMCs, and macrophages [125]. Patients with unstable angina showed higher numbers of CD4+ and CD8+ T-cells producing IFN-γ than patients with stable angina [126]. Together, these results show that T-lymphocyte response through secretion of IFN-γ is important in atherosclerotic disease development.

IL-18 is a pro-inflammatory cytokine that is known to induce IFN-γ activity. Some studies have shown links between this pro-inflammatory cytokine and atherogenesis. When ApoE knockout mice were transfected with IL-18 binding protein (which is the endogenous inhibitor of IL-18), fatty steak development was prevented, and atherosclerotic plaques in the aortic sinus advanced more slowly [127]. In ApoE and IL-18 double knockout mice, the atheromas were significantly smaller and had reduced IFN-γ signaling, although there was increased circulating cholesterol and triglycerides [128]. In humans, carotid intima-media thickness reflected systemic atherosclerosis and was positively correlated with IL-18 concentrations in the blood [129]. Clinically, serum IL-18 levels have been shown to be independent risk predictors for death in patients with coronary atherosclerosis and incidence of coronary events in healthy men [130,131]. 

A myriad of other pro-inflammatory cytokines has been added to the repertoire of atherosclerotic pathophysiology over the last decade. For example, IL-8 is generally considered a chemotactic cytokine and was detectable in the macrophages of plaques and in the circulating monocytes of patients with atherosclerosis, while not in normal donors [132]. IL-8 expression in macrophage foam cells was induced by oxidized LDL loading [133], and IL-8 expression was increased in human arterial atherosclerotic walls. Plasma concentration of IL-8 was associated with higher coronary artery disease risk in healthy men and women [134]. A small clinical study showed IL-8 levels to be higher in patients with unstable atherosclerosis compared to those with stable coronary disease [135]. Another pro-inflammatory cytokine, IL-2, which is secreted by activated T-cells and is a T-cell growth factor, was shown to increase aortic atherosclerotic lesion size when injected into ApoE deficient mice fed an atherogenic diet [136]. Patients with stable angina were observed to have significantly higher IL-2 levels in the plasma than patients with unstable angina, but not different than controls, while IL-2′s soluble receptor was higher in patients with stable angina than those with unstable angina and controls [137]. These results indicate that IL-2 and its soluble receptor play a role in stable plaque development. It is also noted that IL-3, which is released by activated T-lymphocytes, has been shown to induce migration and proliferation of smooth muscle cells in vitro and stimulates vascular endothelial growth factor (VEGF) transcription [138]. These are important steps in the pathogenesis of atherosclerosis concerning the role of this pro-inflammatory cytokine. In another clinical study, IL-7 plasma levels were found to be increased in patients with stable and unstable angina compared to healthy controls and heightened the expression of chemokines in circulating mononuclear cells; IL-7 levels were reduced with aspirin administration [28].

#### 4.1.2. Thrombosis

Thrombosis can occur in the case of atherosclerotic plaque rupture, activating the clotting cascade, and is an important contributor to acute myocardial events. The blood clot can completely obstruct blood flow through the artery, and thus, many pharmacotherapies combating coronary artery disease, such as anticoagulants and anti-platelets, are targeted toward this process. In baboons, TNFα was found to initiate coagulation and fibrinolysis via binding to its p55 receptor (TNFR1) [139]. TNFα and IL-1 were both found to activate endothelial cells, which shifts them to a pro-thrombotic state [140]. In patients with atrial fibrillation, IL-6 levels were higher in patients with increased plasma viscosity, and these patients have an increased risk of stroke [141]. The CANTOS trial also supports the idea that anti-IL-1β therapy could reduce the risk of atherothrombotic disease pathogenesis [115,116].

#### 4.1.3. Acute Myocardial Infarction

Acute myocardial infarction (AMI) is, as the name suggests, an acute event in which the heart muscle begins to die from severe ischemia. Multiple pro-inflammatory cytokines were secreted from nonmyocytes post-infarction in rats [142]. IL-1β increased bone marrow hematopoietic stem cell proliferation and leukocyte production after AMI in ApoE knockout mice, and anti-IL-1β treatment not only reduced this effect but also diminished post-AMI heart failure [143]. Circulating IL-6 and IL-1β levels were found to be higher in patients with AMI compared to stable angina [144]. Cardiac mast cells were degranulated and released TNFα following myocardial ischemia, which was considered to play a role in upregulating IL-6 levels in leukocytes, leading to a cytokine cascade that caused tissue injury [145]. In one study, circulating levels of IL-6 were elevated at admission and 72-h post-AMI compared to controls, and higher levels of IL-6 expression by peripheral blood mononuclear cells at 72-h post-AMI but not at admission were attributed to the activation by macrophage migration inhibitory factor (MIF) [146]. IL-6 was observed to be a strong predictor of 30-day mortality in patients with AMI complicated by cardiogenic shock [147] and was even a predictor of the risk of future MI in apparently healthy men [148]. IL-1β was associated with myocardial dysfunction and non-infarcted left ventricular mass 1-year after ST-elevation myocardial infarction (STEMI) and served as a potential predictor for maladaptive remodeling of the myocardium following AMI and reperfusion [149]. 

In mice, TNFα levels were seen to rise significantly 1-day following AMI, and deletion of TNFα significantly improved myocardial function 3-days post-MI but not at 7 days [150]. This negative effect of TNFα was primarily due to TNFR1, while TNFR2 stimulation was cardioprotective. In male rats with left anterior descending artery ligation, TNFα mRNA and protein production were increased in the myocardium at day 1 and were detectable until day 35 post-AMI. TNFα was detectable not only in the infarcted zone but also in contralateral normal myocardial tissue [151]. In a case-control study, TNFα levels were persistently elevated significantly in patients who developed recurrent coronary events after AMI compared to controls [152]. Soluble TNFR1 was observed to be an independent long-term predictor of death and heart failure in patients with AMI when adjusted for baseline and clinical characteristics [153]. One study found that non-STEMI (NSTEMI) patients have higher levels of TNFα than STEMI patients [154]. Gene enrichment analysis of protein-protein interactions showed AMI patients have enriched IL-8, TNFα, and IL-1β compared to non-AMI patients [155]. Etanercept, a TNFα antagonist, was observed to reduce systemic inflammation but increased platelet-monocyte aggregation in AMI patients and thus was not considered a good candidate for AMI therapeutics [156]. N-acetylcysteine (NAC) administration prevented the rise of TGF-β levels 72 h post-AMI and prevented fibrosis and remodeling of myocardial tissue [157].

#### 4.1.4. Ischemia-Reperfusion Injury

Although reperfusion of ischemic tissue is the gold standard of treatment in clinical practice, ischemia-reperfusion injury (IRI) has been an increasingly apparent unmet need of the healthcare system. IRI is characterized by many complex cellular processes that happen acutely upon reperfusion, including an increased formation of oxygen free radicals, calcium mishandling, and immune activation. These pathways have been observed to result in complications such as myocardial stunning and microvascular dysfunction, often resulting in necrosis [158]. In cultured neonatal rat ventricular myocytes, TNFα was found to increase ROS production and induce mitochondrial DNA damage [159]. This cytokine was released from rat hearts undergoing ischemia-reperfusion (IR) [160,161]. Furthermore, TNFα synthesis was at least partially involved in microvascular transport changes seen in IRI [162]. TNFα knockout mice had reduced infarct sizes following left coronary artery occlusion and improved cardiac function following reperfusion compared to wildtype mice, suggesting that TNFα aggravated IRI via NF-κB activation, which mediates chemokine and adhesion molecule expression and leukocyte infiltration [163]. TNFα also upregulated arginase expression in endothelial cells, which contributes to oxidative stress and endothelial dysfunction in IRI [164]. When given the TNFα inhibitor etanercept before reperfusion, mice had reduced IRI partly via the Notch1 signaling pathway because this agent decreased infarct sizes and improved cardiac function compared to mice with inhibition of the Notch1 pathway [165]. Inhibition of this pathway reduced inducible nitric oxide synthase and enhanced nitric oxide and superoxide production [165]. Anti-TNFα administration at the time of reperfusion reduced superoxide formation and improved coronary dilation in mice, and neutropenic mice had more severe oxidative stress when faced with IRI, indicating TNFα’s deleterious role was neutrophil activation-independent and contributed to oxidative stress and endothelial dysfunction [166]. 

In rat hearts and dogs, anti-TNFα treatment also improved myocardial function during reperfusion [167,168]. Vitexin, a TNFα inhibitor, reduced TNFα and NF-κB levels in IRI in rats [169]. Adenosine, which is also known to attenuate the production of TNFα in macrophages, was also observed to reduce myocardial TNFα production due to IR [170]. Adenosine or TNF-binding protein pre-treatment improved human myocardial tissue function [170]. Interestingly, a low dose of TNFα during ischemia and before reperfusion in isolated rat hearts reduced the infarct area-to-area at-risk ratio while still forming free radicals, suggesting a protective role of TNFα in IRI by inducing cardiac preconditioning [171]. In this context, TNFα has been reported to be produced endogenously during exercise and activates manganese-super oxide dismutase (Mn-SOD) for cardioprotective action against IRI in a diphasic manner in rats [172]. It is pointed out that chronic hypoxia is cardioprotective in IRI, and this protective effect is mediated by TNFR2 [173]. Overall, it appears that TNFα has a dual role in mediating IRI because it is involved in preconditioning and cardioprotection via signaling through its type 2 receptor, and it is produced by ischemic myocardium and induces oxidative stress upon reperfusion via its type 1 receptor.

A closed-chest murine model of IL-6 deficiency showed reduced infarct sizes in IRI compared to wildtype mice, and IL-6 was observed to be important in the early stages of reperfusion injury [174]. On the other hand, another study demonstrated that hypoxia-inducible factor 2 (HIF2) induction of IL-6 expression was cardioprotective in murine IR [175]. These seemingly paradoxical findings reinforce that further research is needed to understand the complex and sometimes pleiotropic nature of cytokines. One component of the inflammation that is induced by IR involves the NLRP inflammasome, which is stimulated by ROS [176]. Inflammasome activation in cardiac fibroblasts, but not cardiomyocytes, was implicated in the initial inflammatory response in IRI and promoted IL-1β expression via caspase-1 [177]. The inhibition of IL-1α in mice with LAD ligation and reperfusion reduced inflammasome formation and infarct size and maintained left ventricular function [178]. NLRP3 activation was observed to mediate the IL-18 released from cardiomyocytes, which in turn activated C-X-C motif chemokine ligand-16 (CXCL16) in vascular endothelial cells to release TNFα, IL-17, and IFN-γ in the heart and trigger ventricular remodeling and cardiac injury [179]. In human atrial myocardium, inhibition of IL-18 with IL-18 binding protein (IL-18BP) or caspase-1 inhibitor improved contractility in IR conditions [180]. Similarly, adding IL-1Ra also reduced cardiac dysfunction, demonstrating a role for IL-1β in IR [180]. Another member from the IL-1 family implicated in IRI is IL-36, which was elevated in murine IRI and aging and was inhibited to improve blood flow and infarct size [181]. Therefore, there are many potential therapeutic targets within the IL-1 family to reduce IRI.

It may be noted that IL-17A has been shown to induce IRI by activating apoptosis of cardiomyocytes and infiltration of neutrophils. In mice with LAD ligation and reperfusion, IL-17A was elevated, and anti-IL-17A treatment improved infarct size and cardiac function and reduced cardiac troponin-T (cTnT) levels [182]. Necrostatin-1 (Nec-1) decreased cardiomyocyte necrosis and inflammatory cell recruitment by inhibiting the Hmgb1-IL-23/IL-17 pathway and attenuated ROS production in IR [183]. IL-1 receptor type 2, which inhibits 1L-1β signaling, was increased in AMI patients following reperfusion, and its overexpression in cardiomyocytes protected from IL-17A-induced apoptosis [184].

### 4.2. Cardiac Remodeling

Most of the prominent cardiac diseases are associated with myocardial remodeling, which is considered to cause heart failure. These changes are generally induced by chronic pressure or volume overload and consist of fibrosis, apoptosis/necrosis, and cardiac hypertrophy. This section is intended to discuss the involvement of pro-inflammatory cytokines in some of the clinical causes and manifestations of the adversely remodeled myocardium, as well as the cellular processes involved in these pathologic transformations. It is also planned to describe the occurrence of adverse remodeling of vascular smooth muscle cells in the pathogenesis of hypertension.

#### 4.2.1. Hypertension

Chronic hypertension is one of the most common modifiable cardiovascular risk factors in the world, which is known to cause mechanical stress on the myocardium [185]. The activation of the renin-angiotensin system (RAS) and elevated sympathetic nervous system activity are classic characteristics of chronic primary hypertension [186]. Dendritic cells and T-cells are important mediators of hypertension and secrete a multitude of pro-inflammatory cytokines [187]. In conditions of high blood pressure, the endothelium is subjected to increased stretch, which affects monocyte differentiation. When human monocytes were cultured with stretched aortic endothelial cells, mRNA levels for many pro-inflammatory cytokines were upregulated, including IL-6, IL-1β and TNFα [188]. The neutralization of IL-6 and hydrogen peroxide production was shown to inhibit intermediate monocyte differentiation in response to endothelial stretch [188]. Angiotensin II was reported to contribute to the development of hypertension and stimulate IL-6 release from human vascular smooth muscle cells [32,189]. Catecholamines were also found to stimulate IL-6 release from endothelial cells [190]. IL-6 treatment increased the expression of epithelial sodium channels in murine kidney cortical collecting duct cells [191], whereas IL-6 knockout mouse studies have shown that IL-6 is an important mediator of angiotensin II and salt-stimulated hypertension [192,193]. Angiotensin II was observed to stimulate IL-6 release via mineralocorticoid receptor activation in humans [194]. In healthy men, plasma IL-6 levels were reported to exhibit a positive correlation with blood pressure [195], whereas treatment of the blood pressure-lowering drug Irbesartan was observed to lead to decreased systolic and diastolic blood pressures in parallel to decreased IL-6 levels in young hypertensive males [196]. Thus, IL-6 has been shown to contribute to hypertension progression, and its levels are depressed by blood pressure-lowering therapies.

The pleiotropic nature of TNFα was highlighted in hypertension. In vitro and in vivo studies have shown that angiotensin II increases the expression of TNFα in renal tissue [197,198]. TNFα knockout mice did not increase the salt and water intake as well as blood pressure in response to angiotensin II infusion, unlike wildtype mice [199]. Spontaneously hypertensive rats fed high fat and high fructose diets were observed to show increased TNFα concentrations along with overactivity of the RAS [200]. T-cell production of TNFα was also stimulated in hypertensive mice, which was prevented by TNFα inhibitor etanercept [201]. Chronic treatment with etanercept prevented hypertension development in fructose-fed rats [202] and mice with systemic lupus erythematosus [203]. In Sprague-Dawley rats fed a high salt diet and infused with angiotensin II for 2 weeks, etanercept was observed to attenuate blood pressure rise and protect against renal injury [204]; however, studies in spontaneously hypertensive rats with high salt intake did not show any effect of etanercept on blood pressure but instead reduced renal inflammation [205,206]. Mice lacking TNFα, specifically in the renal parenchyma, were protected from angiotensin II-induced hypertension and organ damage but increased endothelial nitric oxide synthase expression in kidneys [207]. In fact, renal expression of TNFα was found to contribute to hypertension in Dahl salt-sensitive rats [208]. Together, these animal studies have suggested that TNFα mediates hypertension through renal physiology and sodium homeostasis, and the effect of its inhibition is dependent upon the type of experimented model. It is also pointed out that neural inhibition of TNFα may also play a role in hypertensive physiology [209,210]. In Japanese women, TNFα levels were associated with increased systolic and diastolic blood pressures [211]. Systolic blood pressure was also correlated with TNFα levels in both Canadian men and women with type 2 diabetes [212]. Hypertensive patients in Mongolia given fish oil supplementation had reductions in TNFα levels compared to control corn oil patients and showed a positive correlation with reduced cardiometabolic risk scores [213]. However, further clinical studies on the effect of TNFα inhibition on hypertension are needed in order to understand its clinical therapeutic potential.

Different rodent models of hypertension respond to IFN-γ injection in different ways. In Dahl salt-sensitive rats fed a high salt diet, IFN-γ injection reduced blood pressure and improved renal function, whereas in spontaneously hypertensive rats, IFN-γ administration did not affect blood pressure [214]. In contrast, lack of IFN-γ in mice was associated with a decrease in hypertension in response to angiotensin II infusion [215,216]. On the other hand, in hypertensive chronic kidney disease mice, IFN-γ deficiency did not reduce blood pressure elevation with angiotensin II infusion [207]. In this regard, it should be noted that IFN-γ is necessary for the activation of distal sodium reabsorption and thus interferes with responsive pressure natriuresis in the kidneys [216]. Furthermore, angiotensin II has been shown to induce the accumulation of CD8+ T-cells that produce IFN-γ and the elevation of blood pressure, which was mitigated in mineralocorticoid receptor knockout mice [217]. Excess sodium has also been reported to promote dendritic cell activation, increase T-cell production of IFN-γ, and prime mice for developing hypertension [218]. Thus, variable responses of IFN-γ in different experimental models of hypertension may be related to differences in their pathophysiology for T-cell production and sodium handling status of the kidney tubules. Nonetheless, in patients with hypertensive nephrosclerosis, increased expression of IFN-γ inducible T-cell α chemoattractant was observed in the proximal and distal tubules [219]. In fact, intrarenal IFN-γ was found to regulate angiotensinogen expression in a biphasic manner via Jak/Stat pathways [220], and one study has shown increased IFN-γ-producing T-cells in hypertensive patients in comparison to normotensive ones [221]. Overall, IFN-γ can be seen as an important component of the role of T-cells in hypertensive physiology, but further studies in humans are needed to fully understand its clinical significance.

Human monocytes treated with high salt conditions ex vivo have been shown to stimulate IL-17A production from autologous T-cells [222]. IL-17 treatment for one week was observed to cause increased systolic blood pressure in mice and decreased nitric oxide-dependent aortic relaxation via the RhoA/Rho-kinase pathway [223]. Increased levels of IL-17A in circulation have been shown to result in the remodeling of small mesenteric arteries and increased arterial stiffness in mice, and these changes were considered to contribute to high blood pressure [224]. Since mice deficient in IL-17A were protected from aortic stiffening and IL-17A-induced collagen 3a1 expression, it is likely that this cytokine exacerbated systolic hypertension [225]. IL-17A was also found to affect pressure natriuresis and promote vascular dysfunction in angiotensin-II-induced hypertension [216,226,227]. Mice lacking IL-21, which is produced by T follicular helper cells, showed attenuated hypertension in conjunction with decreased IL-17A levels [228]. Because T-cells producing IL-17A were increased in hypertensive patients compared to normotensive ones [221], it is apparent that IL-17A is associated with hypertension development; however, its clinical significance is not yet clear.

Some studies have examined the relationships between the levels of IL-1 family cytokines and systemic hypertensive physiology. Human monocytes treated with excess sodium had increased expression of IL-1β and NLRP3 inflammasome activation [229]. IL-1β has also been observed to activate vascular smooth muscle constriction in spontaneously hypertensive rats [230]. Interestingly, serum IL-1β levels were higher in patients with essential hypertension compared to patients with familial hypercholesterolemia and healthy controls and were positively correlated with blood pressure in the patients with essential hypertension only [231]. Essential hypertension patients were also reported to have elevated levels of IL-1Ra [232], which potentially could antagonize the effects of IL-1β in these patients [233]. IL-1β inhibition by anakinra, but not canakinumab, was observed to reduce high blood pressure [234,235]. While the usefulness of IL-1β as a therapeutic target for hypertension remains controversial, IL-18 levels in the blood were associated with higher blood pressure [129,236]. The *-137 G/C* polymorphism in the IL-18 gene was associated with increased mean arterial pressure in South African women, and circulating IL-18 levels were independently associated with high blood pressure values [237]. However, inhibition of IL-18 did not significantly alter blood pressure in diabetic patients [238]. Thus, extensive studies are needed to discern the true therapeutic potential of IL-18 targeting in hypertension.

#### 4.2.2. Cardiac Hypertrophy

Cardiac hypertrophy is the compensatory result of a volume and/or pressure-overloaded heart, often associated with hypertension, and leads to heart failure. There are, however, multiple causes for cardiac hypertrophy, all of which are not pathologic in nature, such as exercise. In vitro studies using cardiomyocytes have shown that IL-6 and IL-6R induce cardiac cell hypertrophy [239,240]. Pressure overload-induced cardiac hypertrophy in mice was found to increase myocardial IL-6 and IL-1β levels, although TNFα levels were not affected [241]. Cardiac fibroblasts induced IL-6 signaling in myocytes to cause cardiac hypertrophy via the p38α pathway [242]. Pathological cardiac hypertrophy was also reported to be a classic feature of sickle cell disease, where hemolysis increased IL-6 expression in mice [243]. Mice undergoing excessive exercise training were observed to show signs of pathologic cardiac hypertrophy and increased expression of pro-inflammatory cytokines, including IL-6 [244]. Inhibition of IL-6 with raloxifene in vitro was shown to reduce the cellular hypertrophy induced by IL-6 treatment [245]. These observations are consistent with the view that IL-6 may play an important role in the development of adverse cardiac remodeling.

Another pro-inflammatory cytokine, IL-1, is also known to contribute to cardiac hypertrophy, which is mediated by NLRP3 inflammasome activation [246,247,248]. Left ventricular IL-1β levels were positively correlated with the left ventricle weight/body weight ratio in rats [249]. Overexpression of IL-1 in murine myocardium is enough to cause left ventricular hypertrophy [250]. Mice with IL-1β deficiency were found to have lower heart weight and myocyte size 30 days after aortic banding compared to wildtype mice [251]. Additionally, stretching cardiac fibroblasts was observed to induce low levels of IL-1β secretion; although these levels were insufficient to induce IL-6 production, these were enough to stimulate insulin-like growth factor-1 (IGF-1) for the induction of cardiac hypertrophy [251]. It should also be noted that TGF-β is an important cytokine, which may also be involved in the cardiac remodeling process. In mice treated with angiotensin II, which is known to induce hypertrophy, *TGF-β1* gene expression was reported to be increased for inducing cardiac hypertrophy [252]. Pressure overload was also found to activate TGF-β signaling in cardiac fibroblasts [253]. In TGF-β1 deficient mice, angiotensin II did not cause any increase in left ventricular mass, as seen in wildtype mice [252]. It should be mentioned that TGF-β is primarily secreted by cardiac fibroblasts and has been shown to induce cardiomyocyte hypertrophy in a paracrine manner [254]. The signal transduction system involved in TGF-β-induced cardiac hypertrophy has been demonstrated to be the upregulation of the canonical Wnt/β-catenin pathway [255].

#### 4.2.3. Cardiac Fibrosis

One of the consequences of pro-inflammatory cytokines is tissue repair; however, with limited regenerative capacity, the myocardium has been shown to respond by scar formation [256]. Several immune cells were found to be recruited to the site of injury during this process and contributed to the development of cardiac fibrosis. In the heart, the transition from fibroblast to myofibroblast has been reported to be a key step in this process, as myofibroblasts are known to produce contractile α-smooth muscle actin (α-SMA) and other extracellular matrix (ECM) proteins [257]. It has also been demonstrated that the occurrence of fibrosis is associated with excessive ECM deposition, leading to tissue dysfunction [258]. The expression of multiple pro-inflammatory cytokines post-infarct in cultured rat non-myocyte cardiac cells was also associated with increased collagen production [142]. In vitro, cardiac fibroblast-conditioned media was shown to increase the expression of multiple pro-inflammatory cytokines in cardiomyocytes compared to standard culture conditions [259].

TGF-β is perhaps the most notable cytokine involved in fibrosis. In vascular smooth muscle cells and fibroblasts infected with human TGF-β1 adenovirus, collagen type III gene expression was upregulated [260]. TGF-β stimulated proteoglycan and α-SMA synthesis in cultured myocardial fibroblasts [261,262,263,264]. Mice with TGF-β deleted in the myocardium were found to have decreased cardiac fibrosis and improved survival probability [265]. A disintegrin and metalloproteinase with thrombospondin motifs (ADAMTS)-16 overexpression caused TGF-β activation in cardiac fibroblasts and thus promoted cardiac fibrosis [266]. Wnt/β-catenin pathway signaling increased IL-11 production, which activated TGF-β-mediated fibroblast for transition to myofibroblast [267]. In fact, IL-11 was found to be necessary for the profibrotic action of TGF-β [268]. TGF-β was also demonstrated to play a critical role in the transition between inflammation and fibrosis, as it suppressed inflammation and promoted extracellular matrix deposition [269]. In this context, TGF-β is, therefore, often considered an anti-inflammatory cytokine. Inhibition of TGF-β in mice with aortic constriction was found to result in less fibroblast activation and myocardial fibrosis without any change in cardiac hypertrophy [270]. TGF-β inhibition has been shown to reduce pressure-overload-related cardiac fibrosis in the TAC mouse model of pressure overload [271,272].

Overall, pro-inflammatory cytokines are considered to inhibit fibrotic remodeling of the heart. The addition of IL-1α to cultured fibroblasts was shown to suppress α-SMA production [262]. IL-1β treatment to neonatal and adult rat cardiac fibroblasts in vitro produced selective transcriptional downregulation of fibrillar collagen [273]. IL-1β and TNFα activated specific matrix metalloproteinases in cardiac fibroblasts and degraded collagen, while TGF-β inhibited this effect [273,274,275]. IL-1β also altered the cardiac fibroblast cell cycle in an inhibitory manner [276]. Both angiotensin II and mechanical stress stimulated TNFα production in cardiac fibroblasts but showed no effect in myocytes, whereas isoproterenol was found to inhibit this action [277]. IL-33 was associated with pro-fibrotic signaling in post-MI mice and in patients with heart failure [278,279]. However, in non-ischemic heart failure, IL-33 was cardioprotective against remodeling due to mechanical stress [280]. IL-4 was also implicated as a contributor to cardiac fibrosis [281], as it promoted pro-fibrotic activity in macrophages [282], and IL-17 upregulated remodeling pathways in human cardiac fibroblasts as well as in heart failure in mice [283,284].

Hypoxia-induced mitogenic factor (HIMF) expression in cardiomyocytes was shown to induce IL-6 expression and cardiac fibroblast proliferation, which were inhibited by neutralizing IL-6 with an antibody [239]. IL-6 infusion in rats caused an increase in the stiffness of the ventricle and produced a higher collagen volume fraction [285]. In isolated cardiac fibroblasts, soluble IL-6 receptors and IL-6 were critical in increasing collagen concentration and contributed to the transition to myofibroblasts [285]. The IL-6 trans-signalling pathway was important in aldosterone-induced cardiac fibrosis [286], whereas IL-6 blockade had anti-inflammatory and anti-fibrotic effects in salt-loaded nephrectomised mice [287]. Elabela, an endogenous ligand expressed by cardiac microvascular endothelial cells, was shown to inhibit myocardial remodeling and fibrosis via modulation of the IL-6 signaling pathway [288]. Paradoxically, genetic knockout of IL-6 in mice was found to shift the cardiac cell population and ECM, causing cardiac dysfunction and increases in cardiac fibroblasts [289]. These results suggest that elevated IL-6 levels may promote cardiac fibrosis, but lacking IL-6 from birth could potentiate fibrosis and promote cardiac dysfunction.

#### 4.2.4. Cardiac Apoptosis and Necrosis

Apoptosis is a deliberate programmed cell death response to physical and chemical stress, thereby acting as a protective mechanism in tissues. Apoptosis has been found to be associated with oxidative stress and calcium overload but usually does not cause notable inflammation [290]. Instead, apoptotic cells express molecules to initiate their phagocytosis, which is a clearance mechanism involving the secretion of anti-inflammatory cytokines such as IL-10 [291]. However, when apoptotic cells are not cleared appropriately, an inflammatory cascade occurs to eliminate a potential threat, starting with the migration of innate immune cells and, later, the accumulation of macrophages [290]. Interestingly, TNFα protected adult murine cardiomyocytes against ischemia-induced apoptosis [292], but mice overexpressing TNFα in cardiac myocytes showed myocardial apoptosis [293]. During the course of human AMI, TNFα was not associated with apoptosis but rather IL-8-induced myocardial apoptosis via the activation and accumulation of neutrophils [294]. On the other hand, necrosis is considered a form of accidental cell death that both causes and is caused by inflammation. In necrosis, cells swell, and the plasma membrane becomes disrupted, thus leaking intracellular contents (DAMPs) as well as triggering an inflammatory response. ROS contributes to necrotic cell death by causing mitochondrial damage, and lipoxygenases can also play a role in cell death. Thus far, TNFα has been the primary inflammatory cytokine that is tied to these cell destruction pathways [290]. These pathways are tightly intertwined with cardiac fibrosis, as they involve the balance between tissue repair and cell death. There are still many unknowns in the relationships between apoptosis, necrosis, and inflammation, as this field is ever-evolving, and more research needs to be carried out to fully understand the mechanisms at play.

#### 4.2.5. Heart Failure

The result of several diseases and pathogenic mechanisms discussed so far culminate in heart failure, at which point the heart cannot meet the demands of the body. This section will focus on the role of pro-inflammatory cytokines in affecting cardiac contractility and their role in heart failure in a broader sense to better assess the clinical significance. It should be noted that pro-inflammatory cytokines have been shown to alter contractile protein expression in adult rat cardiomyocytes [295]. Infusion of IL-6 induced diastolic dysfunction in rats [285], which is clinically referred to as heart failure with preserved ejection fraction (HFpEF). HFpEF patients were observed to have higher circulating IL-6 and IL-8 levels compared to patients with asymptomatic hypertension [296], and patients with heart failure had higher IL-6 and TNFα levels compared to healthy individuals [297]. Patients with HFpEF who have reduced TNFα levels due to low-level vagus nerve stimulation showed global longitudinal strain improvement and better quality of life [298]. When HFpEF patients were divided into clinical phenogroups, the most functionally impaired group had high levels of TNFα and tissue remodeling and was at the highest risk of cardiovascular death [299].

Higher IL-6 levels were associated with reduced systolic function in apparently healthy individuals, which perhaps served as a predictive marker for heart failure [300]. Circulating IL-6 levels were associated with increased severity of congestive heart failure (CHF) [297,301,302]. Heart explant specimens from patients with advanced heart failure had higher IL-6 and IL-6R transcript levels than controls, and IL-6 levels were inversely correlated with left ventricular ejection fraction [303]. IL-6 was also reported to play a role in post-MI heart failure and affect adverse remodeling [304]. Coronary sinus samples from patients with CHF had high IL-6 and IL-1β levels compared to peripheral venous blood, indicating that these cytokines are secreted into the blood from heart tissue [305]. The signal transducing receptor subunit glycoprotein 130 (gp130) levels for IL-6 were associated with total and cardiovascular mortality and deaths due to HF [306]. Higher IL-6 levels at 48–72 h were associated with all-cause mortality in patients with acute heart failure at 30 days [307]. Physical exercise training in patients with heart failure was reported to reduce IL-6 and TNFα levels and improve functional status [308,309]. It is also noteworthy that in patients with chronic heart failure, there are macrophages that expressed TNFα, which were not present in control patients [310]. Inhibition of TNFα with adenovirus injection in transgenic mice overexpressing TNFα caused a reversal of the dilated cardiomyopathy seen when given no adenovirus [311]. The use of left ventricular assist devices (LVAD) reduced cardiac TNFα levels [312]. Activin A, a member of the TGF-β superfamily, was also implicated in HF [313], although patients with acute CHF showed lower TGF-β1 than patients without CHF [314].

IL-1β treatment in rat cardiomyocytes reduced the expression of genes related to calcium homeostasis and depressed contractility [315] and affected the responsiveness of calcium channels to beta-adrenergic stimulus [316]. In mice, IL-1β activated IL-18 to induce systolic dysfunction [317], and the IL-18 levels were higher in the myocardium and plasma of patients with CHF [318,319]. Long-term delivery of IL-1 inhibitor anakinra over 12 weeks improved the peak VO_2_ in patients with decompensated systolic heart failure but not in patients with HFpEF [320,321]. Since there are many causes for heart failure, some pro-inflammatory cytokines may have different roles in different diseases. In this regard, it is pointed out that in patients with coronary artery disease-related CHF but not with idiopathic dilated cardiomyopathy-related CHF, TNFα levels were elevated in the plasma along with an atherogenic lipid profile [322]. Similarly, in North India, TNFα and IL-6 levels were found to be higher in patients with ischemia-related CHF compared to patients with valvular and hypertensive heart failure [297].

### 4.3. Arrhythmias

The electrical activity of the heart is key to coordinating its muscular activity. The cardiac remodeling involving hypertrophy and fibrosis affects the electrical pathways and can be seen to predispose the development of arrhythmias. The following section is focused on discussing the role of pro-inflammatory cytokines in the pathogenesis of some common types of arrhythmias.

#### 4.3.1. Atrial Fibrillation

Atrial fibrillation (AFIB) is the most common arrhythmia worldwide, with a lifetime risk of 1/3 to 1/5 in people over the age of 40 [323]. The lifestyle risk factors of AFIB, such as hypertension and ischemic heart disease, all involve structural remodeling and chronic inflammation [323]. Several excellent reviews are available in the literature detailing the pathophysiological relationship between inflammation and AFIB [77,324,325]. The role of pro-inflammatory cytokines in AFIB is evident from the observation that calcium mishandling was shown to be mediated by IL-6 for contribution to the development of AFIB in sterile pericarditis rats [326]. The total collagen in left atrial appendage tissue obtained from patients with AFIB was positively correlated with pro-inflammatory cytokines, including IL-6 and TNFα in epicardial adipose tissue [327]. Both IL-6 and TNFα were associated with increased AFIB risk in the general population, and IL-6 was observed during increased risk of postoperative AFIB [328]. Although in patients with coronary artery disease, IL-6 levels were associated with AFIB, but not TNFα [329], IL-6 levels were higher in AFIB patients than non-AFIB controls [330]. A case-control study showed IL-6, IL-8, and TNFα concentrations were independently seen with AFIB patients compared to controls, and graded TNFα levels were associated with paroxysmal, persistent, and permanent AFIB [331]. Patients with AFIB had higher IL-6 levels during AFIB than during sinus rhythm, indicating an acute response during arrhythmia [332].

It has also been reported that cardiac pacing with metoprolol treatment reduced the levels of TNFα and IL-6 in AFIB patients. Furthermore, IL-6 levels were associated with an increased likelihood of AFIB recurrence after ablation [328] and early recurrence after a short-lasting persistent AFIB with rhythm control [333]. Lower levels of TNFα were seen with increases in response to catheter ablation in AFIB patients [334]. In patients with AFIB, baseline IL-6 and TNFα levels were found to be significant predictors for ischemic stroke [335], and IL-6 levels were higher in AFIB patients at high risk of stroke [141]. Elevated IL-6 levels were associated with the prognosis of mortality and adverse cardiovascular events in anti-coagulated patients with AFIB, even when adjusted for the clinical CHADS2 risk stratification score [336]. The pro-fibrotic marker TGF-β was lower in patients with AFIB, declined over increasing AFIB duration, and was negatively correlated with left atrial diameter [314,330]. Together, these data indicate IL-6 may have a role in AFIB by inducing a pro-thrombotic state, whereas both TNFα and IL-6 are involved in the proinflammatory state that potentiates AFIB.

#### 4.3.2. Other Arrhythmias

There is a lack of studies published about other arrhythmias and pro-inflammatory markers, as they can often be acute events and can result in sudden cardiac death. In atrial flutter patients, the level of IL-6 was higher in peripheral blood than in blood taken from the coronary sinus, indicating a potentially systemic response, with IL-6 level decreasing over time after ablation [337]. Left stellectomy in experimental autoimmune myocarditis rats prevented arrhythmias and reduced IL-6 and TNFα levels [338]. IL-6 has also been shown to be associated with ventricular tachyarrhythmias and an increased risk of sudden cardiac death [339,340,341,342]. IL-18 gene promoter *-137 G/C* polymorphism was also associated with an increased risk of sudden cardiac death in the context of hypertension [343]. It is noteworthy that connexin-43 (Cx-43), a myocardial gap junction protein, has been implicated in ventricular arrhythmogenesis as it was upregulated by TNFα, thus potentially serving a cardioprotective role in preventing arrhythmias [344]. In young people, TNFα levels were elevated in those with ventricular arrhythmias, and it was suggested that TNFα aggravated arrhythmias [345]. On the other hand, IL-1β caused the loss of Cx-43, reduced the coupling of myocytes and myofibroblasts, and was indicated to be involved in the arrhythmias seen post-MI [346]. It is pointed out that TGF-β1 was released by myofibroblasts to induce changes in sodium and potassium ion channels in rats and was suggested to contribute to the electrical remodeling in myocardial injury [347].

### 4.4. Cardiomyopathies

While “cardiomyopathies” is a generic term that refers to a large group of heterogeneous diseases, this section will focus on categories of cardiomyopathies that have not yet been discussed with respect to the role of pro-inflammatory cytokines.

#### 4.4.1. Autoimmune and Inflammatory Cardiomyopathies

It is not surprising that pro-inflammatory cytokines play a role in cardiomyopathies of infectious or autoimmune origin in rheumatic heart disease (RHD), myocarditis, and pericarditis. It is known that RHD is a major cause of cardiovascular morbidity and mortality in young people, particularly those from developing nations [348]. Group A streptococcal infections can lead to an aberrant immune response, resulting in valvular damage in the heart. One of the hallmarks of RHD lesions is the infiltration of T-lymphocytes. RHD rats were found to show significantly higher serum and mitral valve IL-17 and IL-6 levels, which are Th17-related cytokines, than controls [349]. A similar trend was seen in humans, as patients with RHD had higher circulating IL-6, IL-8, IL-2R, and TNFα levels; IL-6 and TNFα levels correlated with valve calcification and functional class severity [350,351,352,353]. Children with acute rheumatic fever had higher IL-8, IL-2, and IL-1β serum levels compared to those with chronic RHD [354,355]. IL-6 levels were higher, and TNFα levels were lower in patients with acute rheumatic fever [356]. IL-4, IL-8, and IL-1RA were observed to predict clinical RHD vs. latent RHD, along with polymorphisms in the IL-2, IL-4, and IL-6 genes [357]. In heart tissue infiltrates from RHD patients, IFN-γ and TNFα expressing Th1 cells were found to be predominant and were considered to contribute to the valvular damage seen in RHD [358]. In patients with RHD-related mitral stenosis who underwent percutaneous mitral commissurotomy, IL-1β, IL-12, IL-6, and IL-4 were decreased [359]. Overall, the cytokines related to Th1 and Th17 cells appear to be important in the infiltration of valvular tissue in the heart in RHD.

Myocarditis is characterized by inflammation in the myocardium and usually follows a viral infection, but also from bacteria, fungi, and parasites. Myocarditis can also be a drug- or chemical-induced abnormality. Eventually, myocarditis has been shown to result in dilated cardiomyopathy. In acute viral myocarditis, natural killer cells have been observed to enter the heart followed by activated T-cells to induce myocardial damage [360]. Mice hearts infected with Coxsackievirus B3 were found to have a primarily Th1-mediated response, with increases in its related cytokines IL-2, IFN-γ, and TNFβ [360]. In mice infected with encephalomyocarditis-induced dilated cardiomyopathy, IFN-γ, TNFα, and IL1β mRNA levels were increased in the heart tissue 3 days after inoculation, peaked at 7 days post-inoculation, and persisted even 80 days later [361]. On the other hand, IFN-γ-deficient mice developed severe and even fatal autoimmune myocarditis, suggesting a protective role of this cytokine [362,363]. IL-12 induced autoimmune pathways independent of IFN-γ signaling and caused the proliferation of Th1 cells [362]. IL-1 and TNF secreted by inflammatory cells in heart infiltrates contributed to postinfectious autoimmune myocarditis [364]. Non-failing hearts were observed to have IL-1 receptor mRNA expression, while heart tissue from patients with inflammatory myocarditis did not show any IL-1 receptor mRNA expression, suggesting receptor downregulation [365]. IL-1α, TNFα, and M-CSF levels were higher in patients with acute myocarditis [366]. Blockade of IL-1 using anakinra prevented myocardial dysfunction in mice [367]. It should also be mentioned that some studies have examined the role of pro-inflammatory cytokines in pericarditis, which is the inflammation of the pericardium lining the heart. Anakinra, the IL-1 inhibitor, was shown to resolve recurrent pericarditis and its associated symptoms [368,369,370]. IL-1 trap rilonacept also rapidly resolved recurrent pericarditis and prevented subsequent episodes [371]. Although these results suggest a role of IL-1 in the exacerbation of pericarditis episodes, the mechanisms remain unclear.

#### 4.4.2. Hypertrophic Cardiomyopathy

Hypertrophic cardiomyopathy (HCM) is the most common inherited genetic heart disease and is the top culprit for sudden death in young people [372]. TGF-β has been implicated in HCM because mutations in sarcomere protein genes and fibrosis are known to contribute to its pathogenesis. In HCM mice, non-myocyte proliferation and fibrosis were observed to be mediated by TGF-β [373]. Mutations in TGF-β inducible early gene-1 (TIEG) were associated with HCM compared to normal hearts [374]. *TGF-β* gene expression and protein levels were elevated in patients with idiopathic HCM myocardium [375,376,377]. Patients with HCM had higher levels of circulating TGF-β than controls, and TGF-β levels correlated with clinical adverse events and hospitalizations [378]. Additionally, high TNFα and IL-2 levels were detected in patients with HCM [366]. In patients with obstructive HCM undergoing nonsurgical septal reduction, TNFα expression was decreased along with regression of cardiac hypertrophy [379]. IL-6, IL-1β, IL-18, and IL-1Ra levels were elevated in serum and heart tissue in patients with HCM [380,381,382,383,384]. Given the genetic etiology of HCM, however, it is difficult to suggest if targeting pro-inflammatory cytokines is a viable therapeutic option for these patients.

#### 4.4.3. Diabetic Cardiomyopathy

Diabetic cardiomyopathy is classically considered as a progression to heart failure in diabetic patients in the absence of coronary artery disease, valvular disease, and hypertension, but this definition has not been universally agreed upon by several investigators [385]. It is thought that myocardial fibrosis is an important contributor to the subsequent diastolic heart failure and arrhythmogenesis that develops and, therefore, must be related to glucose homeostasis and sensing. It is noteworthy that fructose has been observed to increase the expression of TGF-β and collagen markers in vitro, and high fructose feeding in mice was found to induce high expression of multiple pro-inflammatory cytokines, including IL-18, IL-6, IL-1β, and TNFα [386]. TGF-β was shown to stimulate NLRP3 in cardiac fibroblasts [387]. In streptozotocin-induced diabetic rats, the NLRP3 inflammasome was activated and resulted in higher circulating IL-1β and IL-18 levels [388]. Mice with diet-induced diabetes had high IL-1β expression in the left ventricle due to NLRP3 inflammasome activation [389]. Diabetic rat hearts also showed higher expression of TNFα compared to control hearts, which was inhibited by consumption of deep-sea mineral extracts [390]. Macrophage migration inhibitory factor (MIF) was also associated with cardiac dysfunction in diabetic patients [391]. In view of these observations, it is suggested that some pro-inflammatory cytokines may play a critical role in the development of diabetic cardiomyopathy.

## 5. Conclusions

There have been significant advancements in understanding the role of pro-inflammatory cytokines in heart diseases over the past few decades. Despite this progress, it remains unclear whether these cytokines are viable clinical therapeutic targets in cardiac diseases. Particularly, it is noted that inflammation is a key mechanism to protect the body from external insults, and suppressing the key immune regulators could result in detrimental long-term effects. Since several cytokines are involved in several different cardiovascular diseases, it would be difficult to target them in a specific manner. In vitro and in vivo studies allow for more local delivery of pharmacotherapies, but this targeted approach has significantly more practical and logistical challenges in a clinical setting. On the other hand, it would be possible to strike the right balance of anti-inflammatory and pro-inflammatory cytokine pathways, which could lead to improved outcomes in patients. However, extensive studies need to be conducted to make a meaningful conclusion in this regard. Novel technologies may be used to enhance our mechanistic understanding of disease pathogenesis, but it is important to incorporate large patient cohorts that account for heterogeneity in clinical studies, as the field is moving towards precision medicine. These comprehensive data sets are needed in conjunction with new technology to reach clinically-relevant findings. At present, it is unclear whether pro-inflammatory cytokines will become targets for therapies or remain biological markers for mechanistic insights in the pathogenesis of cardiac diseases. It is, however, certain that research in this field is vital and vibrant despite its nuances and complexities.

## Figures and Tables

**Table 1:** Pro-inflammatory and anti-inflammatory cytokines in heart diseases.

Cytokines	Pro- or Anti-Inflammatory	References
Interleukin-6 (IL-6)	Both	[9,10,11,12,13]
Tumor Necrosis Factor α (TNFα)	Pro-inflammatory	[9,10,11,12,14]
Interleukin-1 (IL-1) family		[9,11,14,15,16,17,18,19]
IL-1α	Both	
IL-1β	Pro-inflammatory	
IL-18	Pro-inflammatory	
IL-33	Pro-inflammatory	
IL-37	Anti-inflammatory	
IL-38	Anti-inflammatory	
IL-1Ra	Anti-inflammatory	
Transforming Growth Factor-β (TGF-β)	Both	[20,21]
Interferon-γ (IFN-γ)	Pro-inflammatory	[9]
Granulocyte Colony-Stimulating Factor (G-CSF)	Pro-inflammatory	[22]
Granulocyte Macrophage Colony-Stimulating Factor (GM-CSF)	Pro-inflammatory	[22]
Interleukin-2 (IL-2)	Both	[23,24]
Interleukin-17 (IL-17)	Pro-inflammatory	[25,26]
Interleukin-12 (IL-12)	Both	[7,27]
Interleukin-10 (IL-10)	Anti-inflammatory	[12,15]
Interleukin-4 (IL-4)	Anti-inflammatory	[7,15]
Interleukin-7 (IL-7)	Pro-inflammatory	[28]
Interleukin-21 (IL-21)	Pro-inflammatory	[29]
Interleukin-13 (IL-13)	Anti-inflammatory	[30]
